# Supplementary material for: Metabolic Profiling of SH-SY5Y and Neuro2A Cells in Relation to Fetal Calf Serum (FCS) Concentration in Culture Media
Source: Metabolites. 2024 Mar 26;14(4):188. doi: 10.3390/metabo14040188 (PMC11052363; doi:10.3390/metabo14040188)
Supplement: Supplementary file 1 [file metabolites-14-00188-s001.zip › metabolites-2912205-supplementary.pdf]

Supplementary Material

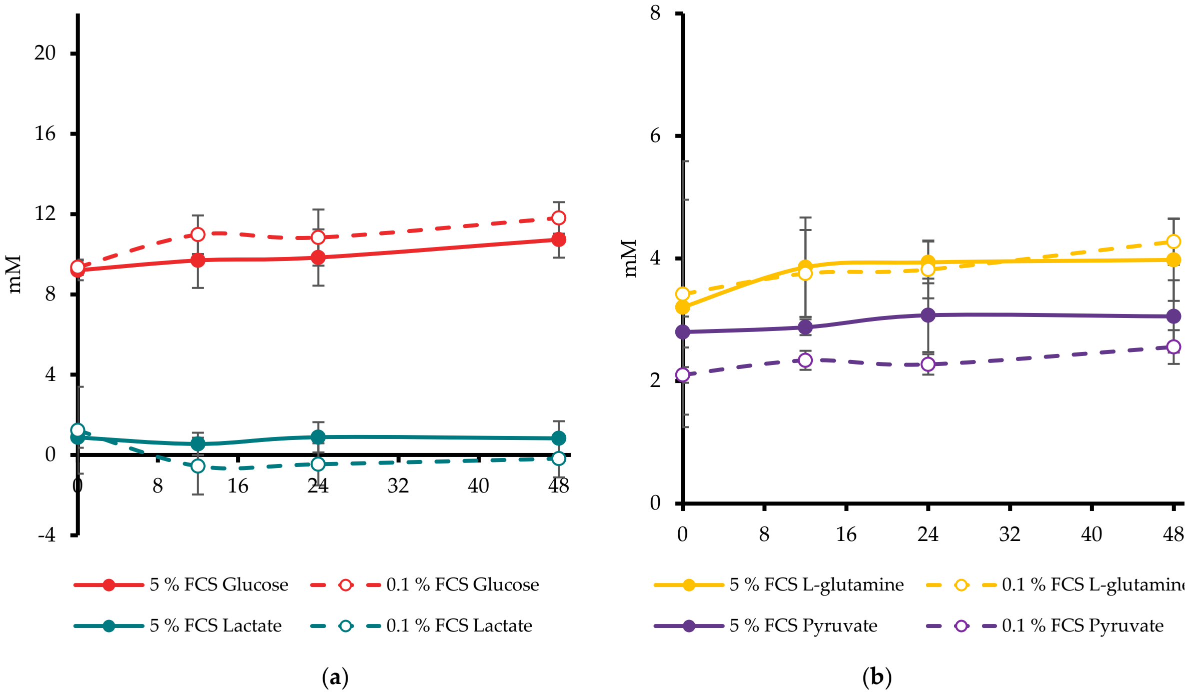

Figure S1. Concentrations of glucose, lactate, pyruvate, and L-glutamine in media of cell-free wells in dependence of FCS concentration. Incubation of the cell-free wells was performed for up to 48 h with DMEM/5% FCS (**a**) or DMEM/0.1% FCS (**b**). Substrate concentration in the media supernatant is shown in mM. n=8. Error bars represent StDev.

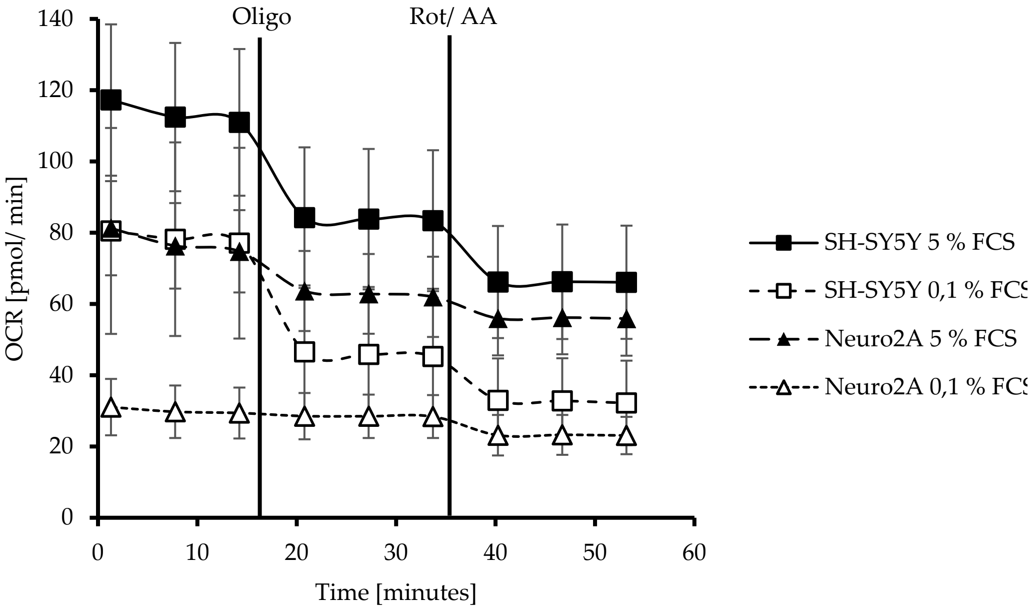

(**a**)

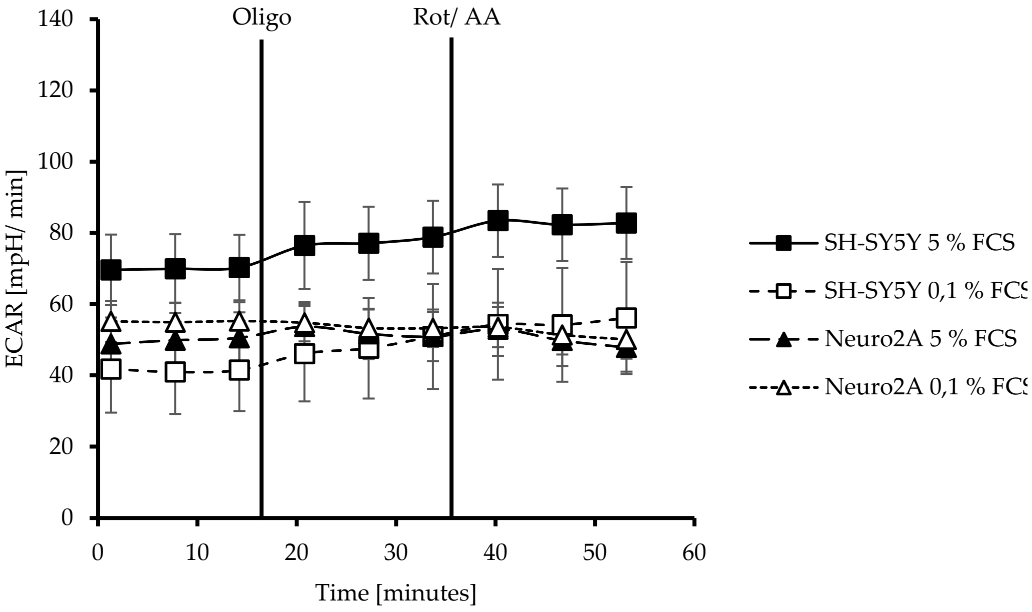

(**b**)

Figure S2. Kinetic curves of OCR in pmol per min (**a**) and ECAR in mpH per min (**b**) measured with Seahorse XF Analyzer utilizing Seahorse XF ATP Rate Assay. After three measurements a final concentration of 1.5 µM Oligomycin (Oligo) was injected and after six measurements 0.5 µM of Rotenone and Antimycin A (Rot/ AA) were injected. n=24. Error bars represent StDev.

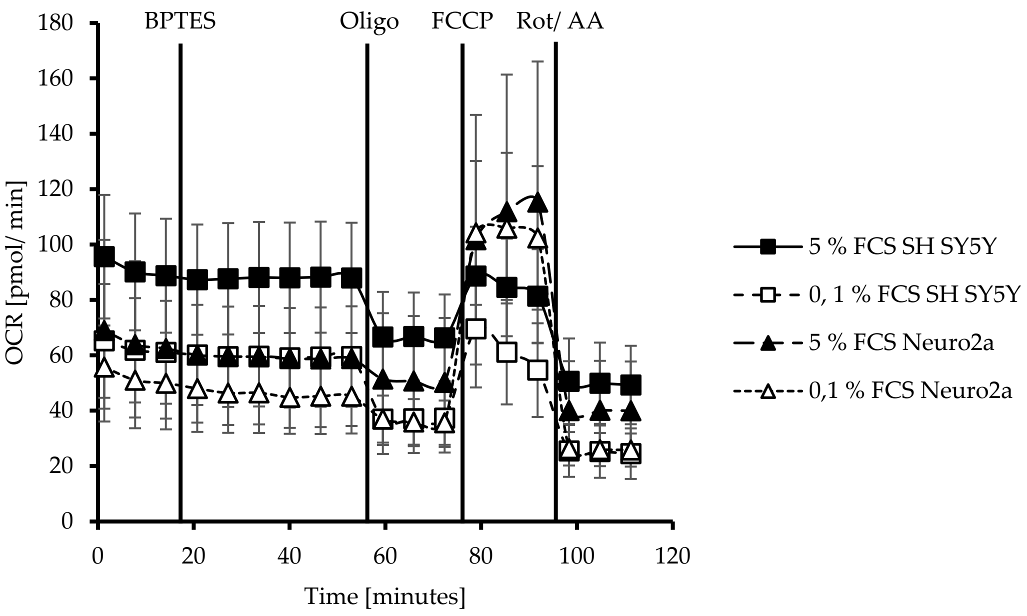

Figure S3. Kinetic curves of OCR in pmol per min measured with Seahorse XF Analyzer utilizing Seahorse XF Glutamine Oxidation Stress Test. After three measurements a final concentration of 3 µM BPTES was injected. Subsequently final concentrations of 1.5 µM Oligomycin (Oligo), 2 µM FCCP and 0.5 µM of Rotenone and Antimycin A (Rot/ AA) were injected. n=24. Error bars represent StDev.
